# HIV prevalence and the cascade of care in five South African correctional facilities

**DOI:** 10.1371/journal.pone.0235178

**Published:** 2020-07-02

**Authors:** Kelsey A. Stevenson, Laura J. Podewils, Vincent K. Zishiri, Kenneth G. Castro, Salome Charalambous

**Affiliations:** 1 Hubert Department of Global Health, Rollins School of Public Health, Emory University, Atlanta, Georgia, United States of America; 2 The Aurum Institute, Johannesburg, South Africa; 3 School of Public Health, University of the Witwatersrand, Johannesburg, South Africa; University of KwaZulu-Natal, SOUTH AFRICA

## Abstract

**Background:**

South Africa is home to the world’s largest HIV epidemic. Throughout the world, incarcerated individuals have a higher prevalence of HIV than the general public, and South Africa has one of the highest rates of incarceration in sub-Saharan Africa. In spite of this, little has been published about the burden of HIV and how care is delivered in South African correctional facilities.

**Objective:**

To estimate the prevalence of people living with HIV and identify initiation and retention in the HIV cascade of care across five correctional facilities.

**Methods:**

Cross-sectional retrospective analysis of 30,571 adult inmates who participated in a tuberculosis screening and HIV counseling and testing campaign in South African correctional facilities (January 1, 2014—January 31, 2015). Descriptive statistics were used to estimate the proportion and 95% confidence intervals of HIV. Proportions of persons retained and lost at each step in the HIV cascade of care under this intervention were calculated. Poisson regression with robust variance estimates were used, and clustering by facility was accounted for in all analyses.

**Results:**

Results of the screening campaign found previously undiagnosed HIV among 13.0% of those consenting to screening, with a total estimated HIV prevalence of 17.7% (n = 3,184, 95% CI: 17.2–18.3%) in the sample. When examining the overall cascade of care, 48.3% of those with HIV initiated care, and overall 45.6% of persons who entered care qualified for ART initiated treatment. A Poisson regression accounting for clustering by facility found HIV high risk groups within the population such as women (aRR = 1.72, 95% CI: 1.57, 1.89), those over 35 years of age (aRR = 2.43, 95% CI: 1.53, 3.85), and people incarcerated less than one year (aRR = 1.41, 95% CI: 1.19, 1.67).

**Conclusion:**

In this setting, routine screening is recommended, and measures are needed to ensure that persons diagnosed are adequately linked to and retained in care.

## Introduction

South Africa is home to the world’s largest HIV epidemic, with an estimated 7 million people living with HIV and 180,000 deaths due to AIDS in 2015 [1, 2]. South Africa also has one of the highest tuberculosis (TB) incidence rates in the world (834 per 100,000), and an estimated 73% of TB patients in South Africa are co-infected with HIV [3].

Throughout the world, incarcerated individuals tend to have a higher prevalence of HIV than the general public [4]. This increased HIV prevalence is often exacerbated by high rates of other infectious diseases, such as TB, and an increased incidence of high risk behaviors among inmates [4]. Limited data suggest these global trends are mirrored in the sub-Saharan African region, with HIV prevalence among inmate populations being greater than that found among non-incarcerated populations [4, 5].

The rate of incarceration in South Africa is among the highest in the world, estimated at 413 per 100,000 [3, 6], making understanding the status of the HIV epidemic in the context of South African correctional facilities especially important. A 2014 systematic review of the HIV cascade of care in low- and middle-income settings demonstrates a relative dearth of information [7], and limited data available on the status of screening, diagnosis, treatment, and management (the cascade of care) for HIV in correctional settings throughout the region [8].

Improved data on the status of HIV in correctional facilities is key for informing programmatic planning, policy development, and implementation [9]. Any failure to provide or ensure screening, continuity of care, and treatment to individuals throughout incarceration, and following release, constitutes a violation of inmates’ human rights [10]. Correctional services have a unique opportunity to engage individuals with a high prevalence of HIV risk behaviors in HIV screening and treatment [4]. This analysis intends to contribute to a more nuanced understanding of the state of HIV screening and the cascade of care, the burden of HIV, and factors associated with HIV in the context of five South African correctional facilities.

### HIV testing and care algorithms in South African correctional facilities

The South Africa Department of Health has developed fairly comprehensive guidelines for the management of HIV, TB, and sexually-transmitted infections (STIs) within correctional facilities [11]. These guidelines recommend that voluntary HIV counseling and testing (HCT) be offered to all inmates at entry, during incarceration, as per request by inmate, as part of routine screening campaigns, as part of integrated primary health care services, and upon release. The spectrum of engagement in the HIV cascade of care consists of screening for HIV, followed by linkage to care, commencement of antiretroviral treatment (ART), retention in care, adherence to ART, and eventual viral suppression and maintenance [12]. ART initiation guidelines in place at the time of data collection for this analysis recommended ART initiation for individuals with a CD4+ result less than 350 cells/μL, as well as for those with a TB diagnosis [11].

The objectives of the current study are to: 1) estimate the prevalence of HIV overall, and by facility; 2) examine associations between clinical and sociodemographic factors and HIV; and 3) examine practices for the HIV cascade of care under a programmatic intervention.

## Methods

### Study population

The current analysis utilized data collected by a South African non-governmental organization as part of a programmatic intervention to conduct enhanced TB screening and HCT at five correctional facilities located in Gauteng, Mpumalanga, Limpopo, and North West provinces in South Africa [13]. All inmates aged 18 or older in the facilities between January 1, 2014 and January 31, 2015 were eligible for inclusion in this analysis (N = 30,571). Inmates, for the purpose of this analysis, refers to both individuals that are sentenced and those awaiting sentencing. All inmates had the same access to health services whether or not they participated in the screening campaign.

Facility-specific populations were calculated using the number of inmates aged 18 and above recorded at each site during the enhanced screening intervention. All inmates were screened for TB but could opt out of receiving HCT. Patients were considered eligible for HCT if they did not have a documented HIV diagnosis prior to the outset of the screening campaign. Analyses examining HIV prevalence and the HIV cascade of care were restricted to those consenting to HCT and those known to be living with HIV prior to the beginning of the campaign.

### Data collection

Demographic and symptom variables were collected via self-report, while laboratory results, HIV diagnosis, and treatment initiation were abstracted from the electronic laboratory database TrakCare and patient charts. Department of Correctional Service (DCS) records were used to determine duration of incarceration.

All persons consenting to HCT provided informed consent and had a finger-prick blood sample collected and screened for HIV using the UniGold (Trinity Biotech) point-of-care rapid HIV test kit. Screening tests with an HIV-positive result were confirmed using the ABON HIV 1/2/O Tri-Line Human Immunodeficiency Virus Rapid Test Device (ABON Biopharm) point-of-care rapid test kit. In the case of discordant results between the screening and confirmatory results, a venous blood sample was collected for enzyme-linked immunosorbent assay (ELISA) and the ELISA result was interpreted as the final HIV test outcome.

All inmates were assessed for the presence of key symptoms for TB, including cough lasting two or more weeks, fever, night sweats, and weight loss. All inmates also underwent digital chest x-ray (CXR). Those with suspected TB, on the basis of a positive symptom screen or abnormal CXR result, were asked to provide a sputum sample for further TB investigation by GeneXpert *Mycobacterium tuberculosis* (MTB)/rifampicin (Rif) (Xpert; Cepheid) testing. Those with suspected TB were also advised to undergo HCT.

Per South African DCS guidelines, any patient newly diagnosed with HIV is to be assessed for active TB infection, via symptom screening, history of exposure, physical examination, and CXR. Patients newly diagnosed with HIV are also to be assessed for latent TB infection using a tuberculin skin test, where 5mm induration is considered positive [11].

### Data analysis

Prior to analysis data was deduplicated on the basis of prisoner number, screening enrolment date, age, gender, and facility. A separate temporary dataset of potential duplicates was generated and examined. True duplicates were removed from the analysis, and in the case of records matching on the aforementioned variables but varying in completeness of the record, the most relevant record to the analysis was retained, on the basis of a scoring variable. The scoring variable was developed by assigning value to variables of interest, such as diagnosis of HIV or TB, presence of HIV testing data, and symptom report. Among duplicate records, the record with highest score was retained.

An exploratory analysis was then conducted in order to determine whether outliers or illogical values existed in the dataset. When these were encountered, they were set to missing. As it is not possible to return to the source data, any values not easily validated were set to missing due to the level of uncertainty when they could not be triangulated or imputed by other data. Descriptive statistics were used to estimate the proportion and 95% confidence intervals (CI) of HIV infection, both overall and by facility. The prevalence of HIV infection was calculated using the total number of inmates at each facility consenting to HCT and inmates known to be living with HIV prior to the commencement of the HIV screening campaign.

Potential associations between demographic and clinical factors and HIV were initially evaluated by bivariate analysis and restricted to inmates consenting to HCT and those known to be living with HIV prior to the start of the campaign.

Following the bivariate analyses, factors demonstrating a significant association based on a p-value less than or equal to 0.2, factors previously demonstrated to be significant in the literature, or biologically plausible factors were included in the final Poisson regression to examine their associations and overall effect on the outcome of HIV. Poisson regression with robust variance estimates were used, as HIV was a common outcome (>10%), and Poisson regression with robust variance estimates provides a more reliable estimate of prevalence risk ratio compared to standard logistic regression. Clustering by facility was accounted for in all analyses. The final model contained independent factors that had an association with the outcome of interest at an alpha level of ≤0.05. The majority of the analyses were conducted using SAS (Version 9.4. 2013), with the Poisson regression was conducted using Stata Statistical Software (Release 15 2017).

All inmates with a confirmed positive HIV result, whether newly diagnosed, or diagnosed prior to this campaign, were considered having entered into the cascade of care. Inmates living with HIV that received CD4+ testing were considered, for the purposes of this analysis, to be linked to care, as presence of CD4+ testing while incarcerated indicated that an inmate had received care relating to HIV beyond their participation in an HCT campaign, and in the case of a diagnosis made prior to their incarceration, demonstrated engagement with HIV-related care while incarcerated. Under the 2013 South African Department of Health guidelines, inmates with CD4+ results less than 350 cells/μL, as well as inmates living with HIV that had also been diagnosed with TB, were considered eligible for ART initiation [11].

### Ethical review

The original study protocol and research instruments were reviewed and approved by the Human Research Ethics Committee at the University of Witwatersrand (Johannesburg, South Africa) and Emory University Institutional Review Board (Atlanta, Georgia). The research protocol was also reviewed and approved by the U.S. Centers for Disease Control and Prevention (Atlanta, Georgia).

## Results

### Study population

A total of 30,571 participants across five facilities (2,985 to 11,613 inmates per facility) were screened for TB between January 1, 2014 and January 31, 2015 (Table 1). The majority of the population (N = 30,571) were male (94.5%; n = 28,894), and nearly half (45.8%; n = 14,016) were between 25 and 34 years of age. Most of the inmates who incarcerated at the time of this campaign were black African (94.3%; n = 28,818), and 70.3% (21,488) had been incarcerated for less than a year.

**Table 1:** Characteristics of persons included in analysis of HIV in five South African correctional facilities (N = 30,571).

Variables	HIV n (%)*	Total (%)
**Facility Size**		
Small (<4,000)	729 (22.9)	9,159 (30.0)
Large (≥4,000)	2,455 (77.1)	21,412 (70.0)
Missing	0 (0.0)	0 (0.0)
**Gender**		
Male	2,890 (90.8)	28,894 (94.5)
Female	291 (9.1)	1,632 (5.3)
Missing	3 (0.1)	45 (0.1)
**Age (years)**		
18–24	368 (11.6)	6,142 (20.1)
25–34	1,553 (48.8)	14,016 (45.8)
35–44	753 (23.6)	5,243 (17.2)
44–54	183 (5.7)	1,529 (5.0)
55+	316 (9.9)	3,541 (11.6)
Missing	11 (0.3)	100 (0.3)
**Race/Ethnicity**		
Black/African	3,114 (97.8)	28,818 (94.3)
Mixed race	40 (1.3)	977 (3.2)
Indian/Asian	6 (0.2)	150 (0.5)
White/European	17 (0.5)	532 (1.7)
Other	4 (0.1)	49 (0.2)
Missing	3 (0.1)	45 (0.1)
**Duration of incarceration**		
Less than 1 year	2,516 (79.0)	21,488 (70.3)
1–2 years	275 (8.6)	3,562 (11.7)
3–4 years	99 (3.1)	1,720 (5.6)
5–9 years	117 (3.7)	1,748 (5.7)
10 years or more	64 (2.0)	926 (3.0)
Missing	113 (3.5)	1127 (3.7)
**Tuberculosis Disease Identified During Screening**		
Positive	33 (1.0)	144 (0.5)
Negative	3,151 (99.0)	30,427 (99.5)
Missing	0 (0.0)	0 (0.0)

*Denominator comprised of those known to be living with HIV prior to the campaign, and those with positive HIV results found through the screening campaign (n = 3,184).

While the HIV screening denominator for this analysis is comprised of the 967 individuals diagnosed with HIV prior to the start of the screening program, and the 16,991 individuals that consented to HCT (n = 17,958), the HIV data presented in Table 1 is out of the 967 people diagnosed with HIV prior to the start of the screening program and the 2,217 people newly diagnosed with HIV through the program (n = 3,184).

### HIV screening and cascade of care

**Fig 1:**
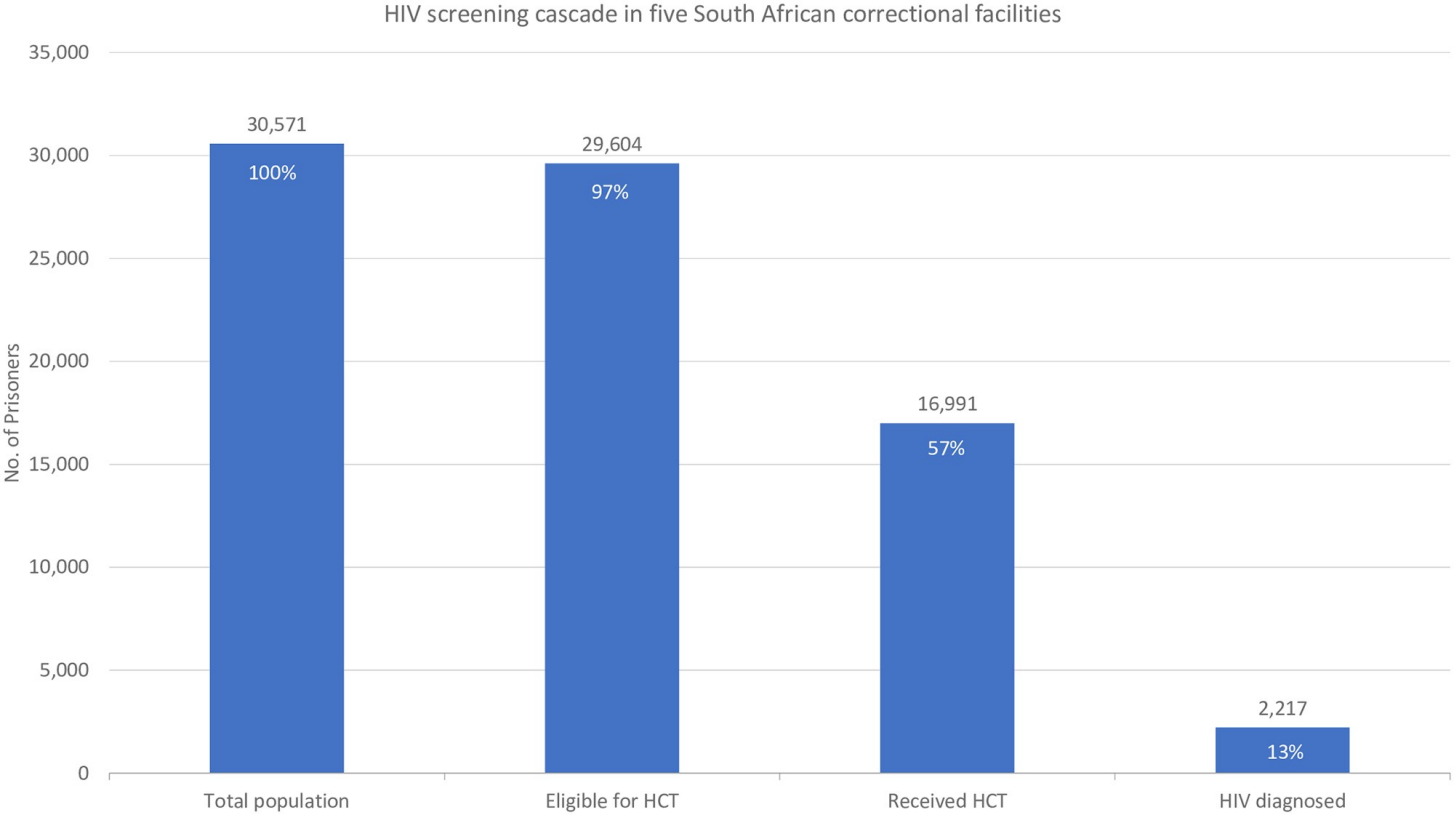
HIV screening cascade in five South African correctional facilities.

Overall, when excluding the 967 inmates diagnosed with HIV prior to the start of the program, 96.8% (29,604) of the sample of inmates were eligible for HCT (Fig 1). Of these, 57.4% (16,991) consented to HIV screening during the parent study. 2,217 persons (13.0% of those that received HCT) were newly diagnosed with HIV through this program.

**Fig 2:**
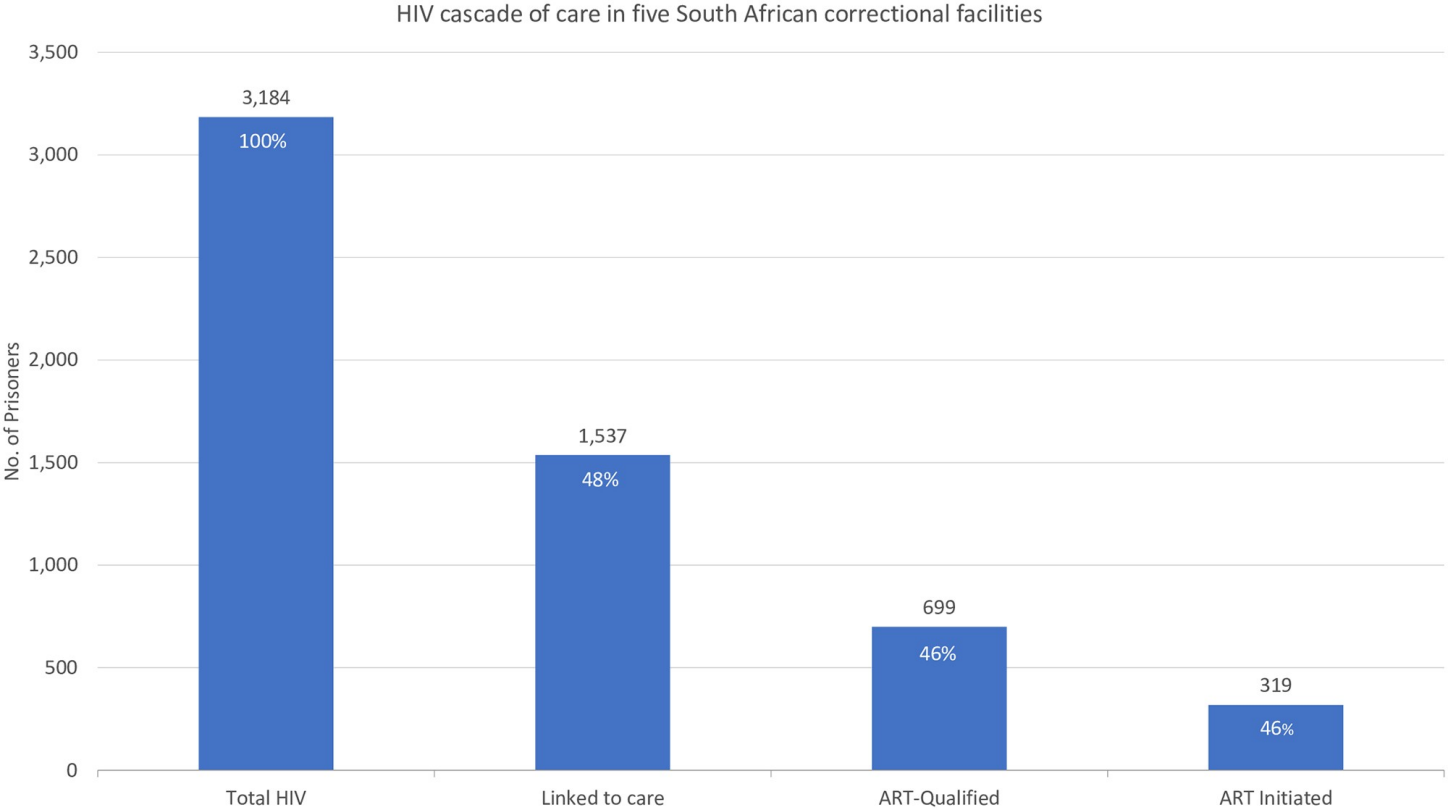
HIV cascade of care in five South African correctional facilities.

Of the total known HIV-positive population (n = 3,184), 48.3% (n = 1,537) had CD4+ cell count results and were considered linked to care (Fig 2). 699 inmates (45.5% of people living with HIV) met the criteria for ART initiation per Department of Health guidelines at the time of this screening campaign (by either having a CD4+ result less than 350 cells/μL, being found to have TB, or both), and 45.6% (n = 319) of those eligible had ART initiation documented. Approximately half of all eligible inmates were lost at each observed step in the aggregate cascade.

### Associations between HIV and demographic and clinical factors

Demographic and clinical factors associated with HIV infection were not found to meaningfully differ between those diagnosed prior to incarceration, or those diagnosed through HCT offered at facilities. Female inmates had a 72% higher risk of HIV infection compared to male inmates (aRR: 1.72, 95% CI: 1.57, 1.89, p < .001) (Table 2). Inmates 25 years old or older were more likely than younger inmates to be living with HIV, as were those incarcerated less than one year. The risk of HIV infection was over three times greater among black inmates than among inmates of all other races (aRR: 3.44, 95% CI: 2.83, 4.19, p < .001).

**Table 2:** Factors associated with HIV among individuals in five South African correctional facilities (n = 17,958).

Variables	Bivariate Relative Risk (95% CI)	Adjusted Relative Risk (95% CI)
**Gender**		
Male	Reference	Reference
Female	1.60 (1.45, 1.78)	1.72 (1.57, 1.89)
**Age (years)**		
18–24	Reference	Reference
25–34	1.98 (1.78, 2.20)	2.10 (1.48, 2.97)
35+	2.16 (1.93, 2.40)	2.43 (1.53, 3.85)
**Race/Ethnicity**		
All other races/ethnicities	Reference	Reference
Black/African	2.99 (2.36, 3.77)	3.44 (2.83, 4.19)
**Duration of Incarceration**		
Less than 1 year	1.27 (1.08, 1.48)	1.41 (1.19, 1.67)
1–2 years	2.11 (1.88, 2.37)	2.38 (1.80, 3.17)
3+ years	Reference	Reference

### HIV prevalence and proportion

The overall prevalence of HIV in the study population, including those with both known HIV and those consenting to HCT, was 17.7% (n = 3,184, 95% CI: 17.2–18.3%), but facility prevalence varied between 8.6% and 21.4% (Table 3). Across all five facilities, 33 inmates were known to be living with HIV and were diagnosed with TB disease at the time of the screening (0.1% of the total population).

**Table 3:** HIV prevalence by correctional facility.

	Total Population	Previously Diagnosed HIV	HCT Uptake	Newly Diagnosed	Total HIV Prevalence
**Site**		No. (%)	No. (%)	No. (%)	No. (%)
A	9799	427 (4.4)	5,294 (54.0)	495 (9.4)	922 (16.1)
B	11,613	366 (3.2)	6,795 (58.5)	1,167 (17.2)	1,533 (21.4)
C	3,074	44 (1.4)	1,320 (42.9)	177 (13.4)	221 (16.2)
D	3,100	35 (1.1)	1,350 (43.5)	84 (6.2)	119 (8.6)
E	2,985	95 (3.2)	2,232 (74.8)	294 (13.2)	389 (16.7)
**Total**	**30,571**	**967 (3.2)**	**16,991 (55.6)**	**2,217 (13.0)**	**3,184 (17.7)**

### Stratified cascades

In order to gain a more nuanced understanding of factors impacting linkage to care and treatment initiation, cascades were stratified on the basis of facility size, duration of incarceration, and age.

**Fig 3:**
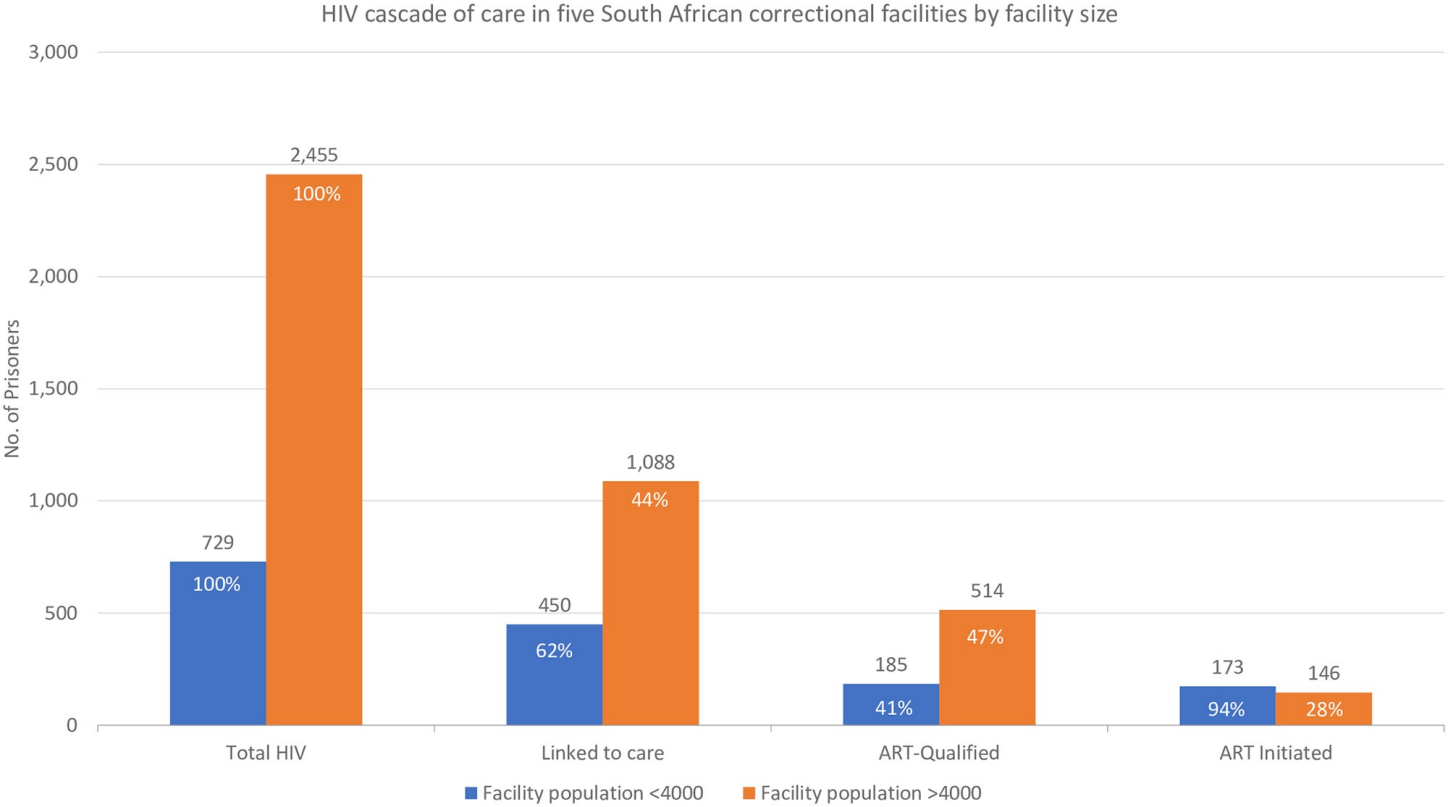
HIV cascade of care in five South African correctional facilities by correctional facility size.

In smaller facilities, over half (61.7%) of people living with HIV were linked to care, and 93.5% of ART-qualified inmates had initiated ART (Fig 3). Larger facilities saw similar linkage to care (44.3%), but a lower percentage of those qualified initiating ART (28.4%).

**Fig 4:**
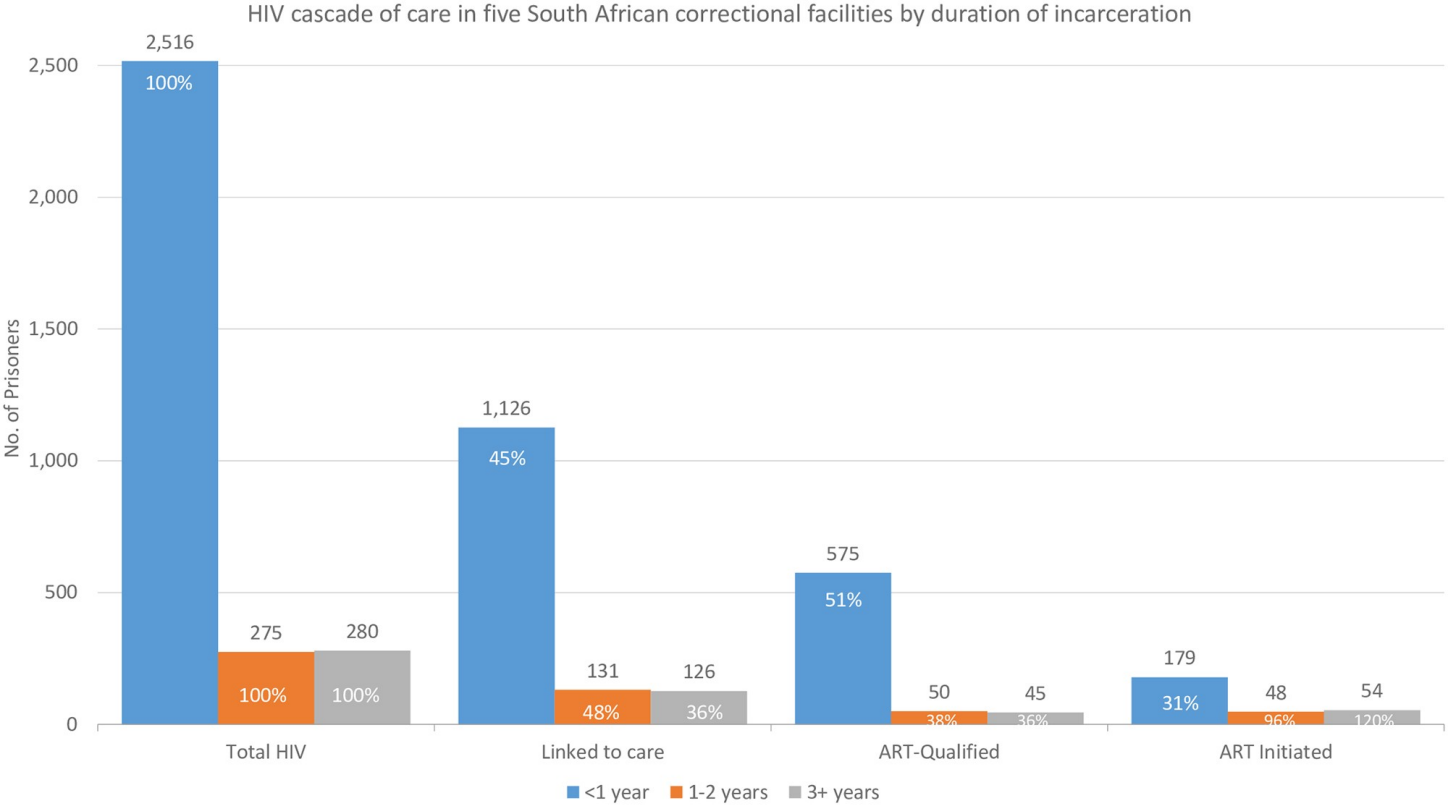
HIV cascade of care in five South African correctional facilities by duration of incarceration.

Linkage to care occurred at similar proportions among inmates incarcerated for any length of time, while ART initiation occurred at higher proportions among those incarcerated for over one year (Fig 4). A greater proportion of inmates incarcerated less than one year were ART-qualified (51.1%) than inmates incarcerated over one year, with 38.2% of those incarcerated one to two years, and 35.7% of those incarcerated three or more years qualified for ART.

**Fig 5:**
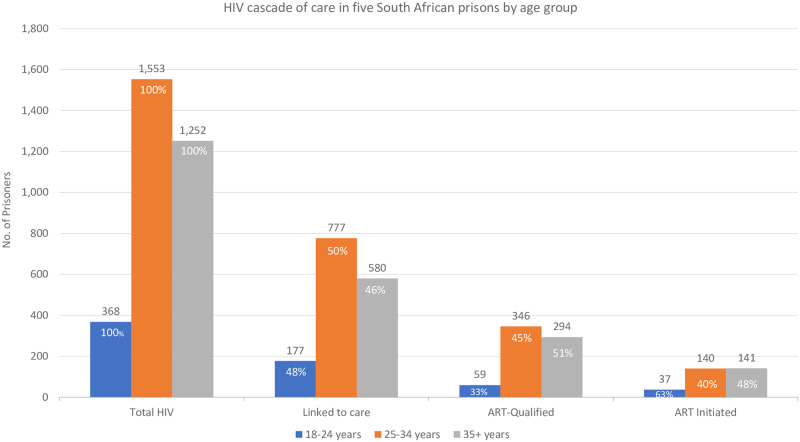
HIV cascade of care in five South African correctional facilities by age group.

Linkage to care was approximately 50% for all age groups, and the lowest proportion of ART-qualified persons was observed among inmates age 18 to 24 years (33.3%) (Fig 5). ART initiation was similar among inmates 25 to 34 (40.5%) and inmates age 35 or older (48.0%), but highest among those 18 to 24 (62.7%).

## Discussion

The overall prevalence in this sample is lower than the 2015 South African general population’s HIV prevalence of 19.2% among people 15 to 49 years of age [1], and lower than the findings of a 2012 paper that described a 29.6% prevalence in one South African prison [14]. It is possible that intensive investment of resources within DCS and beyond has resulted in a lowered HIV prevalence and incidence, as has been demonstrated in other settings [15, 16], though given that the majority of HIV diagnoses in the study population were unknown prior to this screening campaign, it is unlikely that the lower prevalence compared to the general population is the result of such an investment. While over half of all HCT-eligible inmates elected to partake in testing, it is unknown whether HIV prevalence among those that did not partake in HCT is comparable to those that did, though it is possible that inmates that refused testing are more likely to be HIV positive, which is a potential explanation for why we found the HIV prevalence to be lower in this study population than in the South African general population.

Women in the sample had over double the risk of living with HIV that men did, which is reflected by the literature available on the nature of the epidemic in South Africa, in which women experience a higher HIV prevalence compared to men, and the general population [1, 17, 18, 19]. The age group with the highest prevalence of HIV was inmates 35 to 54 which stands in contrast to previous examinations of demographic characteristics associated with HIV in South African prisons, where those under age 25 demonstrated the highest HIV prevalence [8]. While the number of inmates known to be living with both HIV and TB was low (n = 33), the connection between HIV and TB has been well established [3, 5, 8, 9], and underscores the importance of ensuring persons with TB disease are prioritized for routine HIV screening.

While HCT uptake varies widely between demographic groups in sub-Saharan African countries, home-based HCT programs in South Africa and elsewhere in the region have attained uptake as high as 98% [20, 21, 22]. HIV-related stigma is a known barrier to HCT uptake in South Africa [23, 24], but during this screening campaign over half of all eligible inmates opted to partake in HCT (57.4%). Both aggregate and stratified cascades demonstrated a loss in the proportion of qualified patients at most steps. This attrition is well-documented in the HIV cascade of care; even in non-correctional settings [12].

Staff shortages are one of the many challenges to providing routine testing in South African correctional facilities [23, 24]. Frequently, inmates who failed to enter into HIV care after testing lacked access to adequate point of care CD4+ testing due to health care staff shortages. Lack of integration between information health systems often resulted in missing or delayed care and made monitoring care and treatment challenging [25]. At the time of this study there was no functional mechanism to link and trace inmates in HIV care post-release from incarceration, and frequently those that dropped out of HIV care were inmates that had been transferred to or from another facility. This attrition must be addressed, as delayed entry into care has a known impact on morbidity and mortality rates, increased health system costs, and increased drug resistance and development of complicated HIV [26, 27, 28].

Throughout sub-Saharan Africa, rates of linkage to care following HIV diagnosis have varied between 17% to 78%, and among those ART eligible, ART uptake ranged from 14% to 95% [29], emphasizing the need for both the Department of Health and DCS to implement efforts that bolster HIV services across the cascade of care for all inmates.

The extent to which referrals for post-release care were being made was unable to be assessed in the current analysis, as eligibility for post-release referrals was not included in this dataset. Due to reports of high levels of turnover and large numbers of remand inmates [30], we may expect that a larger proportion of those who were HIV-positive were released from incarceration during the time of the evaluation than were documented as having a post-release referral to care. Ensuring sustained engagement in care beyond incarceration must remain a priority if improvements in care made during incarceration are to be maintained.

Universal test and treat (UTT) has since been implemented in South Africa and in the Department of Corrections. Recent evidence has demonstrated that in spite of human resources constraints and poor coordination between health information systems, the existing delivery and monitoring systems for HIV care and treatment within DCS are capable of supporting sustained UTT [31]. Evidence also suggests that transient populations and those with high mobility, such as those in prison, dilute the impact of UTT interventions [32, 33].

There is a lack of data on whether or how the size of a correctional facility may influence retention or loss from the HIV cascade of care. This analysis found that while linkage to care was comparable in smaller and larger facilities, the vast majority (93.5%) of ART-qualified inmates had initiated treatment in smaller facilities. In larger facilities, less than one third (28.4%) of qualified inmates had initiated treatment. It is conceivable that many of those incarcerated for less than one year had not yet been sentenced, which increases the likelihood of transfer from one facility to another and is a possible explanation for the lower treatment initiation among this subpopulation. A 2012 systematic review of ART initiation in sub-Saharan Africa found older age to be associated with treatment initiation [34], but in this analysis, just under half of those aged 35 or older had initiated treatment.

### Limitations

Programmatic data was used for this analysis, and as this data was not intended for research purposes, it required fairly intensive cleaning. The deduplication process introduced a conservative bias to the sample by erring towards maintaining a more complete record. During this externally-supported screening campaign, all inmates were screened for TB, and HCT uptake was over 50%, but it is uncertain how reflective this is of routine practice within DCS. It is possible that HIV prevalence may be underestimated, due to 41.3% (n = 12,395) of the eligible population not consenting to HCT. While TB screening was compulsory, inmates had the option to opt out of HCT, resulting in the analysis of HIV and HIV-related variables being restricted to inmates who underwent HCT or were known to be living with HIV at the outset of the campaign. The data accessible for this analysis did not allow for the evaluation of all stages of the cascade of care, including retention in care and viral suppression. Missing information included meaningful viral load data, cotrimaxazole initiation, crytpococcal screening, and indication of eligibility for release. The analysis examining cascades of care was also dependent upon recorded values in the patient record and laboratory. It is possible there were parts of care that were carried out, but not captured. Symptoms of TB were taken as part of a clinical history and exam and were based on self-report, making them subject to forgetfulness or falsification. TB was also diagnosed on the basis of GeneXpert, rather than culture, which could underestimate prevalence, though combining the use of GeneXpert and CXR helps mitigate this risk. While these findings are important, the conclusions drawn from the data are limited as significant changes in global and national policy regarding HIV testing and treatment have occurred since the data were collected. While literature on precisely how and to what degree UTT may improve initiation and retention in the HIV cascade is still scarce, there is evidence that the 2015 WHO guidelines to treat persons regardless of CD4+ cell count can be successfully implemented (32, 33).

### Strengths

The generalizability of this analysis to correctional facilities outside of South Africa may be limited; however, based on available demographic information from DCS, it does appear that the analysis is generalizable to facilities in South Africa [34]. The strength of this analysis is further bolstered by the sample of over 30,000 incarcerated persons, and the inclusion data from multiple facilities. This examination of HIV screening and the cascade of care in these five South African correctional facilities has the potential to help directly guide DCS program activities by identifying sub-populations most at-risk for HIV and attrition from the cascade of care.

## Recommendations and conclusion

DCS and the South African Department of Health have a unique opportunity to diagnose and treat a population for HIV that may otherwise be difficult to reach. Current guidelines recommend treatment initiation regardless of CD4+ cell count [15], which differs from the guidelines in place at the time of data collection for this analysis [11]. Future research must identify how UTT has affected both linkage to care and treatment initiation within DCS facilities. Improving linkage to care, especially for inmates at larger facilities, is of key importance, as is strengthening treatment initiation among inmates at larger facilities, and inmates incarcerated less than one year. The externally-supported screening campaigns that formed the basis for this analysis found a significant amount of previously-undiagnosed HIV, underscoring both the value and necessity of offering routine screening upon entry and throughout incarceration. As the majority of those with HIV were incarcerated for less than a year, screening upon entry for HIV is especially important.

The period of incarceration provides a vital opportunity for identifying previously undiagnosed cases and initiating treatment, particularly given the recent shift to UTT [4, 35, 36] DCS has the potential to significantly bolster engagement with the HIV cascade of care, and should work to find innovate ways to serve in that role [35].

Associations discovered in this analysis provide a means of better identifying inmates with the greatest risk of HIV, allowing for more targeted screening efforts, resulting in more identified cases and the opportunity to intervene in the progression of illness. By targeting the identified high-risk sub-populations for enhanced screening and linkage to care, DCS could make a substantial impact on improving not only the health of inmates, but the broader community.
